# The Impact of Vitamin D Deficiency on Gestational Diabetes Mellitus Risk: A Retrospective Study

**DOI:** 10.7759/cureus.65037

**Published:** 2024-07-21

**Authors:** Ramona E Dragomir, Daniela E Gheoca Mutu, Romina M Sima, Oana D Toader, Ruxandra V Stănculescu

**Affiliations:** 1 Doctoral School, Carol Davila University of Medicine and Pharmacy, Bucharest, ROU; 2 Department of Anatomy, Carol Davila University of Medicine and Pharmacy, Bucharest, ROU; 3 Clinical Department of Plastic and Aesthetic Surgery and Reconstructive Microsurgery, Prof. Dr. Agrippa Ionescu Clinical Emergency Hospital, Bucharest, ROU; 4 Department of Obstetrics and Gynecology, Carol Davila University of Medicine and Pharmacy, Bucharest, ROU; 5 Department of Obstetrics and Gynecology, Alessandrescu-Rusescu National Institute for Mother and Child Health, Polizu Hospital, Bucharest, ROU

**Keywords:** 25-hydroxyvitamin d, insulin resistance, pregnancy, gestational diabetes mellitus, vitamin d deficiency

## Abstract

Background

Nowadays, there has been growing attention to vitamin D deficiency's impact on women of reproductive age, both during pregnancy and the preconception period. Because of its possible impact on female reproductive ability, vitamin D deficiency may have multiple implications for maternal and fetal health. Currently, the correlation between vitamin D deficiency and gestational diabetes mellitus (GDM) is gaining attention, mainly due to contradictory findings in the literature.

Methods

We conducted a single-center, retrospective study involving data from 106 pregnant women to establish a possible link between vitamin D deficiency and GDM risk. We analyzed variables such as vitamin D status, the occurrence of gestational diabetes, and body mass index to identify significant statistical correlations among them.

Results

Within our study group, the average vitamin D level was 19.5 ± 7.8 ng/mL. Regarding vitamin D status, 59 (55.7%) pregnant women had vitamin D deficiency, 36 (34%) had vitamin D insufficiency, and 11 (10.4%) had optimal vitamin D levels. GDM was diagnosed in 18 cases, representing 17%. After the statistical analysis, we found a positive correlation between gestational diabetes and vitamin D deficiency (chi-square = 4.472, p = 0.049). However, we did not find a significant correlation between gestational diabetes and optimal vitamin D status.

Conclusions

Pregnant women with vitamin D deficiency are at an increased risk of developing GDM and may benefit from vitamin D supplementation. We believe that further research is necessary to have a comprehensive understanding of vitamin D's effects on pregnancy.

## Introduction

The increased prevalence of vitamin D deficiency across all life stages has emerged as an important health issue mainly because of its association with numerous adverse health outcomes, including complications in pregnancy [1,2]. Current research indicates a high incidence among pregnant women globally [3-6]. In addition to its well-documented impacts, such as reduced calcium levels and nutritional rickets, current research has found a connection between vitamin D deficiency and reproductive issues in women, including an increased risk of pregnancy-related conditions [7,8]. These conditions include gestational diabetes mellitus (GDM), preeclampsia, and a higher incidence of spontaneous abortion, birth by cesarean section, and preterm births [9-15]. In neonates, vitamin D deficiency has been associated with an elevated risk of developing conditions such as type 1 diabetes mellitus, bronchiolitis, asthma, autism, and multiple sclerosis [6,16]. Therefore, attaining and sustaining the optimal vitamin D status through supplementation before conception and during pregnancy may reduce the risk of maternal and fetal complications and diseases in newborns and infants [6].

Vitamin D is crucial for the proper functioning of the immune system, cell growth modulation, and inflammation regulation. Maintaining adequate vitamin D levels during pregnancy is vital for ensuring optimal health outcomes for both the mother and fetus. The active form of vitamin D, 1,25-dihydroxyvitamin D, plays an important role in regulating various genes essential for immune function and cell differentiation, which are critical processes during pregnancy [3,14].

In addition to its well-known impact on phosphorus-calcium homeostasis and bone health, vitamin D selectively controls the activity of genes related to maintaining the functioning of the cardiovascular system, regulating glucose levels, promoting cell differentiation, and managing the immune response. Several research studies have demonstrated a correlation between vitamin D deficiency and the prevalence of chronic diseases [17,18]. However, meta-analyses on vitamin D supplementation have yielded conflicting results, influenced by variables such as baseline 25-hydroxyvitamin D (25(OH)D) levels, sample size, different vitamin D doses, and adherence to treatment, among other factors [19].

During pregnancy, alterations in carbohydrate metabolism are constant, but their degree and impact on the mother and fetus vary based on individual factors [20]. GDM is the most frequent manifestation of these metabolic changes, particularly in developed countries where obesity prevalence is higher, one of the most significant risk factors [20,21].

Gestational diabetes is defined as any alteration in glucose tolerance with onset or diagnosis during pregnancy [20]. In some cases, it involves the discovery of a prediabetic condition through pregnancy investigations, while in others, it represents the onset of diabetes. It is typically diagnosed at 24-28 weeks of gestation via an oral glucose tolerance test (OGTT) [20]. Similar to type 2 diabetes mellitus, gestational diabetes is characterized by increased insulin resistance by up to 30-40% and reduced insulin secretion in response to hyperglycemia [22]. Placental hormones, such as placental lactogen and placental growth hormone, contribute to increased insulin resistance [23,24]. Pregnancy is considered a state of mild inflammation associated with elevated cytokine levels, such as tumor necrosis factor-alpha (TNF-α), interleukin 6 (IL-6), and C-reactive protein. In obese patients, excess cytokines can induce insulin resistance [25].

Insulin resistance during pregnancy can be influenced by several variables, such as body structure, the occurrence of metabolic syndrome, and other disorders connected to obesity [26]. It is widely known that increased body mass index (BMI) is associated with gestational diabetes [27]. There are also ethnic disparities among healthy adults, children, and adolescents regarding insulin secretion and resistance [28].

Pregnant women with gestational diabetes are at increased risk for hypertension, pre-eclampsia, urinary tract infections, early or late abortion, preterm birth, ketoacidosis, hydramnios, and a higher likelihood of requiring cesarean delivery [29,30]. Gestational diabetes also has significant implications for the fetus/newborn, necessitating careful management to mitigate potential risks. One of the most common complications for the fetus is macrosomia, which can lead to delivery complications, including shoulder dystocia [30]. Newborns of mothers with gestational diabetes are at risk for low blood sugar (hypoglycemia) shortly after birth and respiratory distress syndrome. Studies have also shown that infants born to mothers with gestational diabetes have an increased probability of having obesity throughout infancy or adolescence and then developing type 2 diabetes in adulthood [29].

The mechanisms through which vitamin D deficiency influences the risk of GDM are not yet fully understood [6]. However, it has been demonstrated that 1,25(OH)2D3 affects both beta cell activity and insulin resistance [31-33].

Given the increased incidence of vitamin D deficiency and gestational diabetes, along with their significant effects on both mother and fetus, further research into the association between these two conditions is necessary. Therefore, our research aimed to investigate whether there is a correlation between vitamin D status and gestational diabetes in our study population. Taking into consideration the link between gestational diabetes and obesity, we also analyzed this association in our study group.

Part of this article was previously presented as a meeting abstract and poster at the 21st World Congress of Gynecological and Endocrinology on 8 May 2024.

## Materials and methods

We conducted a single-center, retrospective study in a third-level maternity hospital that included data from 106 pregnant women aged 16-45 years. We performed an in-depth analysis of variables, such as vitamin D status, the presence of gestational diabetes, and BMI, to determine a significant statistical link between them.

Inclusion and exclusion criteria

The participants for our research were chosen based on certain inclusion and exclusion criteria, which are outlined below. First of all, pregnant women who had pre-existing chronic diseases that might independently impact vitamin D levels or pregnancy outcomes were not included in the study [34]. The study only examined singleton pregnancies that reached full term, defined as gestational ages between 37 and 40 weeks. To eliminate regional disparities in vitamin D intake, individuals residing in different geographical areas were excluded. In addition, those taking medical drugs known to disrupt the metabolism of vitamin D were not included. Exclusion was also justified based on the presence of inconsistent medical records. After applying the inclusion and exclusion criteria presented above, we included in our study 106 pregnant women with consistent medical records, which permitted a comprehensive retrospective study.

Data collection

BMI, height, and weight were provided anamnestically by the subjects. The BMI was determined by utilizing the weight (measured in kilograms) and height (measured in meters) obtained at the initial first-trimester appointment. The women were classified into several categories, namely, normal (BMI < 25), overweight (BMI between 25 and 30), or obese (BMI > 30), following the recommendations set by the World Health Organization. Those for whom BMI could not be reliably calculated and those with pre-gestational diabetes were excluded.

Vitamin D serum levels were determined in the first trimester of pregnancy using an internationally approved test. The Elecsys total vitamin D test, which uses a vitamin D-binding protein as the capture protein, was utilized to assess the vitamin D status of the participants [34]. It quantifies the barometer for vitamin D status - total 25-hydroxyvitamin D (25-(OH)D) in serum and plasma using the electrochemiluminescence immunoassay (ECLIA) method. Vitamin D deficiency was defined as levels below 20 ng/mL, vitamin D insufficiency as levels between 20 and 30 ng/mL, and optimal vitamin D levels as above 30 ng/mL, in accordance with the American Institute of Medicine [34].

The diagnosis of GDM was made following local rules, between 24 and 28 weeks of pregnancy, which are in line with the recommendations of the International Association for Diabetes and Pregnancy Study Group. The OGTT was conducted using a 75 g dose of glucose. Glycemia levels were measured at zero hours, one hour, and two hours. Pregnant women were diagnosed with GDM if their glycemia levels surpassed the specified cut-off points at any time during the OGTT. The cut-off criteria for glycemia were as follows: zero hours > 92 mg/dL, one hour > 180 mg/dL, and two hours > 153 mg/dL.

All data utilized were obtained retrospectively from medical records.

Statistical analysis

Results were presented as mean ± standard deviation for continuous variables and as number and percentage for nominal variables. Statistical analysis was performed using SPSS Statistics 29.0 (IBM Corp., Armonk, NY). Chi-squared tests were conducted for nominal variables and logistic regression for continuous variables. A p-value of less than 0.05 was considered statistically significant.

## Results

Our study included 106 pregnant women, of whom 18 (17%) were diagnosed with GDM. The mean BMI was 29.8 ± 5.1 kg/m². Among the participants, 86 (81.1%) were categorized as overweight or obese, with 52 (49.1%) classified specifically as obese. Regarding the presence of obesity and overweight, 17 cases of gestational diabetes (94,4%) were observed in this category.

The average vitamin D level was 19.5 ± 7.8 ng/mL. Within the cohort, 59 (55.7%) subjects had vitamin D deficiency, 36 (34%) had vitamin D insufficiency, and 11 (10.4%) had optimal vitamin D status. Vitamin D deficiency was found in 72.2% cases of GDM and vitamin D insufficiency in 22.2% cases of GDM.

The basic characteristics of the study population, including those with and without GDM, are presented in Table 1.

**Table 1:** Basic characteristics of the study population. GDM: gestational diabetes mellitus; BMI: body mass index.

	All (n = 106)	GDM (n = 18)	No GDM (n = 88)
Vitamin D value (ng/ml)	19.5 ± 7.8	16.4 ± 6.6	20.3 ± 7.8
Vitamin D deficiency	59 (55.7%)	13 (72.2%)	46 (52.3%)
Vitamin D insufficiency	36 (34%)	4 (22.2%)	32 (36.4%)
Optimal vitamin D	11 (10.4%)	1 (5.6%)	10 (11.3%)
BMI (kg/m2)	29.8 ± 5.1	32.2 ± 4.1	29.3 ± 5.2
Obesity	52 (49.1%)	16 (88.8%)	36 (40.1%)
Overweight and obesity	86 (81.1%)	17 (94.4%)	69 (78.4%)

**Figure 1:**
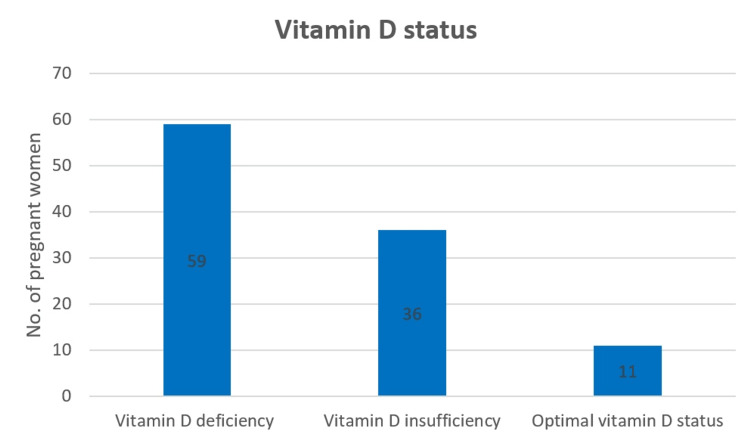
Vitamin D status in the study group.

Regarding vitamin D status in pregnancy, we found that most pregnant women were diagnosed with vitamin D deficiency or insufficiency, as shown in Figure 1. This confirms the idea that vitamin D deficiency is highly prevalent nowadays.

**Figure 2:**
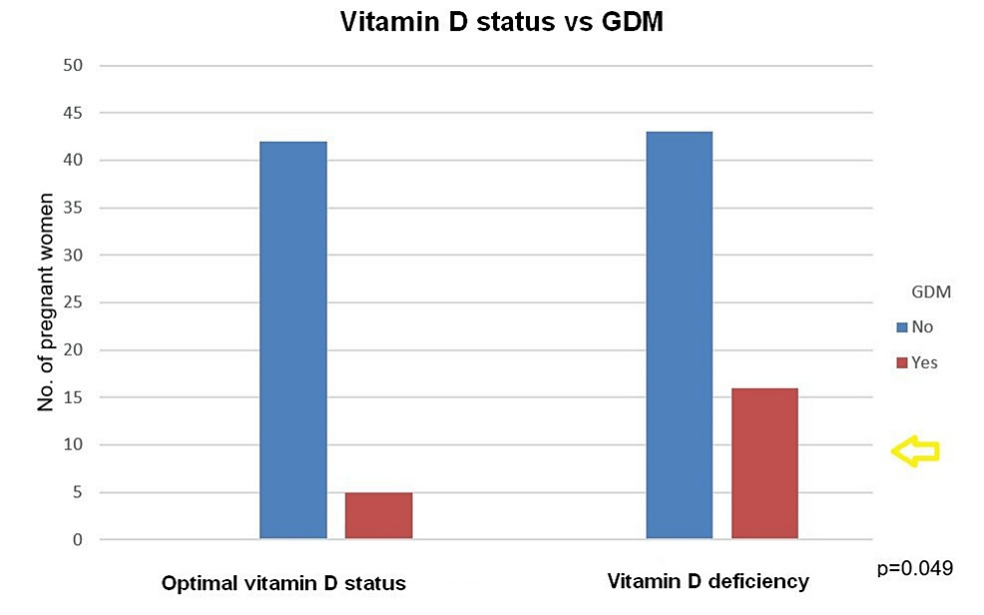
Correlation between vitamin D status and the presence of GDM. GDM: gestational diabetes mellitus.

Vitamin D deficiency was significantly correlated with the presence of GDM (chi-square = 4.472, p = 0.049), as shown in Figure 2. Regarding the statistical analysis between 25-hydroxyvitamin D values and GDM, we found an inverse relationship (OR = 0.913, p = 0.019). Specifically, for each unit increase in 25-hydroxyvitamin D, the odds of having GDM decrease by approximately 8.7%.

Our results showed that optimal 25-hydroxyvitamin D levels were associated with lower GDM rates, although statistical significance was not achieved.

**Figure 3:**
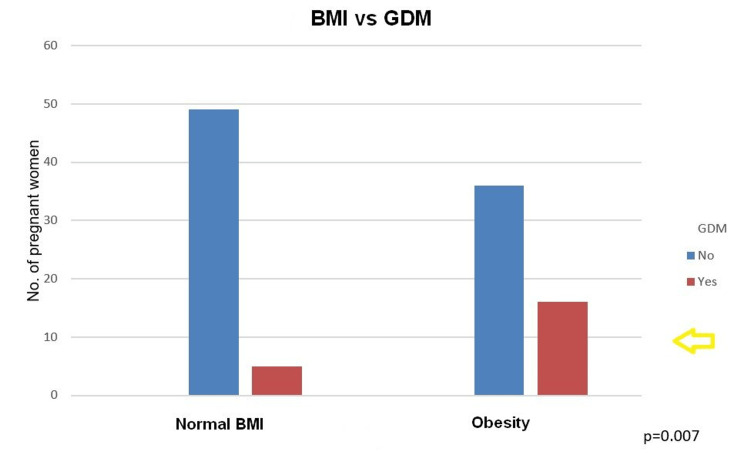
Correlation between BMI and the presence of GDM. GDM: gestational diabetes mellitus; BMI: body mass index.

Obesity significantly increased the odds of GDM (chi-square = 7.715, p = 0.007), and BMI value was directly correlated with the presence of GDM (OR = 1.111, p = 0.029). These results, presented in Figure 3, indicate that pregnant women with obesity have a notably higher likelihood of developing GDM compared to those with normal BMI. Regarding the association between GDM and BMI, we found that for each unit increase in BMI, the odds of having GDM increase by approximately 11.1%.

**Figure 4:**
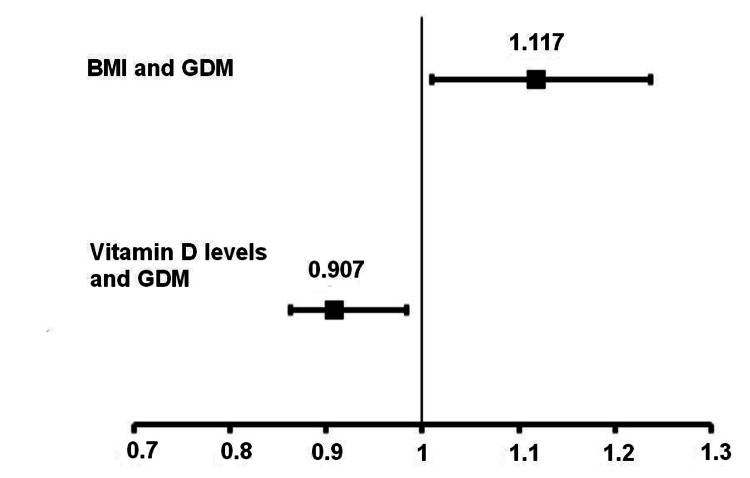
Forest plot - correlation between BMI, vitamin D serum levels, and GDM. GDM: gestational diabetes mellitus; BMI: body mass index.

It is important to note that both vitamin D serum levels (25-hydroxyvitamin D levels) and BMI were significantly correlated with the presence of GDM. We used a forest plot for a better representation of these correlations, as shown in Figure 4.

Neither obesity nor normal BMI significantly correlated with vitamin D deficiency or insufficiency. We found 25-hydroxyvitamin D levels correlated inversely proportionally with BMI but without statistical significance.

## Discussion

GDM and vitamin D deficiency are two common complications during pregnancy, each posing significant risks to both maternal and fetal health. Emerging research suggests a potential link between these conditions, indicating that vitamin D deficiency may contribute to the development of gestational diabetes and exacerbate its effects. Understanding the relationship between these conditions is crucial for improving pregnancy outcomes.

The global prevalence of gestational diabetes is challenging to assess due to varying diagnostic criteria and population characteristics. Reports indicate that 7-14%, or even up to 25%, of pregnant women may develop gestational diabetes. In the United States, gestational diabetes complicates approximately 14% of pregnancies, reducing fetal viability and increasing the likelihood of cesarean delivery [33]. In addition, it increases the likelihood of the mother developing type 2 diabetes in the future and increases the fetus's risk of congenital and anatomical defects, such as macrosomia, birth trauma, and respiratory distress syndrome [33,35]. Table 2 presents the main complications related to gestational diabetes during pregnancy [33-35].

**Table 2:** Gestational diabetes complications in pregnancy.

Pregnant women	Fetus/newborn
Hypertension and preeclampsia	Macrosomia
Infection	Hypoglycemia
Cesarean delivery	Respiratory distress syndrome
Polyhydramnios	Preterm birth
Increased risk of type 2 diabetes later in life	Obesity and type 2 diabetes later in life

Pregnancy-related vitamin D deficiency is becoming increasingly concerning because of its possible effects on the health of the fetus and the mother. Vitamin D, an essential nutrient, plays a significant role in calcium homeostasis and bone metabolism, but its importance extends beyond these functions.

The relationship between vitamin D deficiency, placental function, and gestational diabetes is complex and multifaceted. Vitamin D deficiency can impair pancreatic β-cell function and the immune system, leading to insulin resistance and the development of this pathology [32,33]. It was shown that pancreatic beta cells contain both vitamin D receptors and the 1-alpha-hydroxylase enzyme, which is responsible for converting inactive vitamin D into its active form [31]. Any alteration regarding vitamin D status can impact the adequate function of pancreatic β-cell function, decreasing insulin secretion in response to glucose [31,32]. Furthermore, vitamin D's role in modulating inflammation and immune responses is critical in placental health. Inflammatory conditions in the placenta, such as those seen in preeclampsia, may be exacerbated by vitamin D deficiency, potentially contributing to the development of gestational diabetes [24,30].

The impact of vitamin D deficiency and supplementation on the development of gestational diabetes remains a subject of debate. Several studies have found significant correlations between vitamin D deficiency and the presence of gestational diabetes [36-38]. Studies suggest that maintaining an optimal vitamin D status throughout pregnancy could enhance placental function, improve insulin sensitivity, and decrease the likelihood of GDM [39]. Studies on vitamin D supplementation during pregnancy have explored its potential to lower the incidence of gestational diabetes and improve maternal and fetal outcomes. It was reported that vitamin D supplementation during pregnancy (up to 4000 IU daily) can reduce the rate of gestational diabetes or its complications [40-42], primarily by lowering insulin resistance levels [33,40].

A prospective study measured vitamin D levels in women at 16 weeks of gestation. Among these women, 57 later developed gestational diabetes, while 114 remained unaffected by this condition. The study found that serum vitamin D levels were lower in those who developed gestational diabetes [43]. Additionally, another study from Canada concluded that vitamin D deficiency in the early weeks of pregnancy is a risk factor for gestational diabetes. Others have failed to find similar associations [42,44,45].

Numerous studies have reported an inverse relationship between serum levels of 25(OH)D and the risk of developing GDM; however, a consensus has yet to be reached [46]. Sadeghian et al. found that for every 4 ng/mL increase in circulating 25(OH)D, the risk of gestational diabetes decreased by 2%, with an overall risk reduction of 29% for those in the highest 25(OH)D level category compared to those in the lowest level category [6,47]. One meta-analysis reported a significant positive correlation between vitamin D status and the risk of gestational diabetes [48,49], while another showed that vitamin D deficiency increased the risk of gestational diabetes by 45% [50]. Palacios et al. discovered that administering vitamin D supplements during pregnancy decreases the likelihood of GDM [6,51]. Additional studies have evaluated vitamin D supplementation in pregnancy with already-established gestational diabetes. Wang et al. conducted a meta-analysis that concluded that vitamin D supplementation has an important role in decreasing circulating glucose levels and insulin resistance among pregnant women with gestational diabetes [52].

Other studies have investigated the correlation between vitamin D status and the occurrence of gestational diabetes. Zhang et al. discovered a positive correlation between low levels of vitamin D in pregnant women and an increased risk of developing gestational diabetes, indicating that vitamin D deficiency may impair insulin function and contribute to the onset of this pathology [43]. Burris et al. studied the impact of vitamin D status on inflammation and the immune system and noted that vitamin D deficiency could exacerbate inflammatory responses, potentially leading to insulin resistance and gestational diabetes [53].

It is also important to mention that the association between vitamin D levels and gestational diabetes can be bidirectional. Gestational diabetes has been shown to increase the activity of CYP24A1 in the trophoblast, which metabolizes both 25(OH)D and 1,25(OH)2D to their inactive forms [36]. Therefore, a significant association between vitamin D deficiency and gestational diabetes might not imply a one-way cause-effect relationship.

However, we have not found significant results concerning women who were found to have optimal vitamin D levels. This could be explained by the relatively small study population and an even smaller number of cases with optimal vitamin D levels.

Another important aspect is the association between gestational diabetes and increased BMI [54]. It is known that higher levels of insulin resistance are correlated with elevated BMI [54]. Insulin resistance typically increases throughout pregnancy to guarantee adequate glucose provision to the developing fetus. Nevertheless, in pregnant women with a higher BMI, the pre-existing insulin resistance may exacerbate, causing the pancreatic beta cells to be unable to adequately respond by producing more insulin [54]. As a consequence, GDM occurs. Our study reconfirmed the association between obesity and GDM. Additionally, BMI was positively correlated with the odds of gestational diabetes in a linear fashion, consistent with the findings of a recent systematic review [54].

While most data suggest that vitamin D deficiency increases the risk of gestational diabetes, a consensus has yet to be reached. One of the possible reasons we also encountered is the difficulty in choosing appropriate cut-off levels for vitamin D deficiency and optimal levels. For the optimal value, we chose the vitamin D level above which parathyroid hormone concentration plateaus is 30 ng/mL [55]. However, it is unclear whether the non-musculoskeletal benefits of vitamin D also plateau at this level or if higher levels are required [56-58]. Moreover, the available data primarily apply to non-pregnant adults, and it is uncertain if they are applicable during pregnancy. For vitamin D deficiency, we used the value recommended by the American Institute of Medicine and the European Food Safety Authority, which is 20 ng/mL [59,60].

Nonetheless, our research has some possible limitations due to its retrospective nature and relatively small population size. Additionally, being a single-center study might affect the general applicability of the results to the general population.

Several limitations may affect the results of studies on this subject, including ours as presented above, and could account for the discrepancies between different research. These limitations primarily involve a lack of standardization in vitamin D level cut-offs, timing (gestational age) of vitamin D determination, vitamin D supplementation dosage, and the form of vitamin D (D2 vs. D3) used [61-66].

## Conclusions

Recently, the impact of vitamin D deficiency on pregnancy, including its correlation with gestational diabetes, has garnered increased attention. In our study, vitamin D deficiency was associated with the incidence of gestational diabetes. Regarding optimal vitamin D status and gestational diabetes, no statistically significant correlation was found. Vitamin D supplementation before and during pregnancy may help reduce the risk of pregnancy-related conditions, including the risk of gestational diabetes.

Since vitamin D deficiency represents a growing global health issue, this area of research requires further investigation and large-scale randomized clinical trials to better understand its implications.
